# Combatting antimicrobial resistance via the cysteine biosynthesis pathway in bacterial pathogens

**DOI:** 10.1042/BSR20220368

**Published:** 2022-10-07

**Authors:** Joanna L. Hicks, Keely E.A. Oldham, Jack McGarvie, Emma J. Walker

**Affiliations:** 1 Te Huataki Waiora, School of Health, University of Waikato, Hamilton, New Zealand; 2 Te Aka Matuatua, School of Science, University of Waikato, Hamilton, New Zealand

**Keywords:** CysE, CysK, CysM, cysteine synthesis, O-acetylserine sulfhydrylase, serine acetyltransferase

## Abstract

Antibiotics are the cornerstone of modern medicine and agriculture, and rising antibiotic resistance is one the biggest threats to global health and food security. Identifying new and different druggable targets for the development of new antibiotics is absolutely crucial to overcome resistance. Adjuvant strategies that either enhance the activity of existing antibiotics or improve clearance by the host immune system provide another mechanism to combat antibiotic resistance. Targeting a combination of essential and non-essential enzymes that play key roles in bacterial metabolism is a promising strategy to develop new antimicrobials and adjuvants, respectively. The enzymatic synthesis of L-cysteine is one such strategy. Cysteine plays a key role in proteins and is crucial for the synthesis of many biomolecules important for defense against the host immune system. Cysteine synthesis is a two-step process, catalyzed by two enzymes. Serine acetyltransferase (CysE) catalyzes the first step to synthesize the pathway intermediate *O*-acetylserine, and *O*-acetylserine sulfhydrylase (CysK/CysM) catalyzes the second step using sulfide or thiosulfate to produce cysteine. Disruption of the cysteine biosynthesis pathway results in dysregulated sulfur metabolism, altering the redox state of the cell leading to decreased fitness, enhanced susceptibility to oxidative stress and increased sensitivity to antibiotics. In this review, we summarize the structure and mechanism of characterized CysE and CysK/CysM enzymes from a variety of bacterial pathogens, and the evidence that support targeting these enzymes for the development of new antimicrobials or antibiotic adjuvants. In addition, we explore and compare compounds identified thus far that target these enzymes.

## Introduction

Antibiotic resistance is a slow burning global pandemic that threatens not just human health and life expectancy but also food production. The discovery of antibiotics in the 1920s and 30s was a game changer for human health and agriculture. These drugs have saved millions of lives from previously fatal infections and massively reduced risk from surgical interventions. Within agriculture, antibiotics have been used for animal health and, in healthy food-producing animals to promote growth and prevent disease. Yet not 100 years after the ‘golden era’ of antibiotic discovery we are facing the problem of extensively drug resistant (XDR) and multidrug resistant (MDR) strains of bacterial pathogens. The rising emergence of antibiotic resistance and the lack of new antibiotic classes discovered over the past 60 years requires new strategies to overcome resistance and target bacterial pathogens. Identifying novel druggable targets different from those currently targeted by antimicrobials are crucial to overcoming antibiotic resistance. Along with this, adjuvant strategies targeting nonessential targets such as those important for bacterial virulence, persistence or host colonization are gaining interest [1,2]. Targeting these enzymes that are nonessential during the normal bacterial life cycle but become essential during infection and host invasion, could decrease the incidence of antimicrobial resistance (AMR), as inhibition of these nonessential targets would facilitate clearance by the immune system without stimulating resistance. Targeting nonessential enzymes often decreases bacterial fitness, thereby inhibitors of these enzymes could act as adjuvants to enhance the potency of existing antibiotics. Most pathogens spend at least part of their life cycle in an extremely challenging environment; infection and survival within the hostile host environment relies on a series of sulfur-containing molecules, including Fe-S clusters, thiamine, thioredoxin, glutathione and biotin, which have detoxifying capabilities and reducing power [3,4].

Cysteine is absolutely crucial for the synthesis of sulfur-containing biomolecules; therefore, inhibiting cysteine synthesis is a promising strategy for both potential new antimicrobials and antimicrobial adjuvants. Inhibition of cysteine biosynthesis has been proven to interfere with a pathogen’s ability to fight oxidative stress, infect the host and establish long-term infection [5–7]. For example, cysteine metabolism is a promising drug target in *Salmonella enterica* serovar Typhimurium [8–10] and *Mycobacterium tuberculosis* [7,11,12] where suppression or reduction of cysteine synthesis led to decreased fitness and infectivity. Inhibition of cysteine biosynthesis has also been associated with a dysregulated oxidative stress response, enhancing the antimicrobial activity of existing antibiotics [6,9,10]. Mammals lack the biosynthetic machinery for the *de novo* synthesis of cysteine from inorganic sulfur, relying on the reverse transulfuration of dietary methionine to obtain cysteine. Whereas bacteria and plants have highly conserved enzymes for the assimilation of inorganic sulfur into cysteine [13].

**Figure 1:**
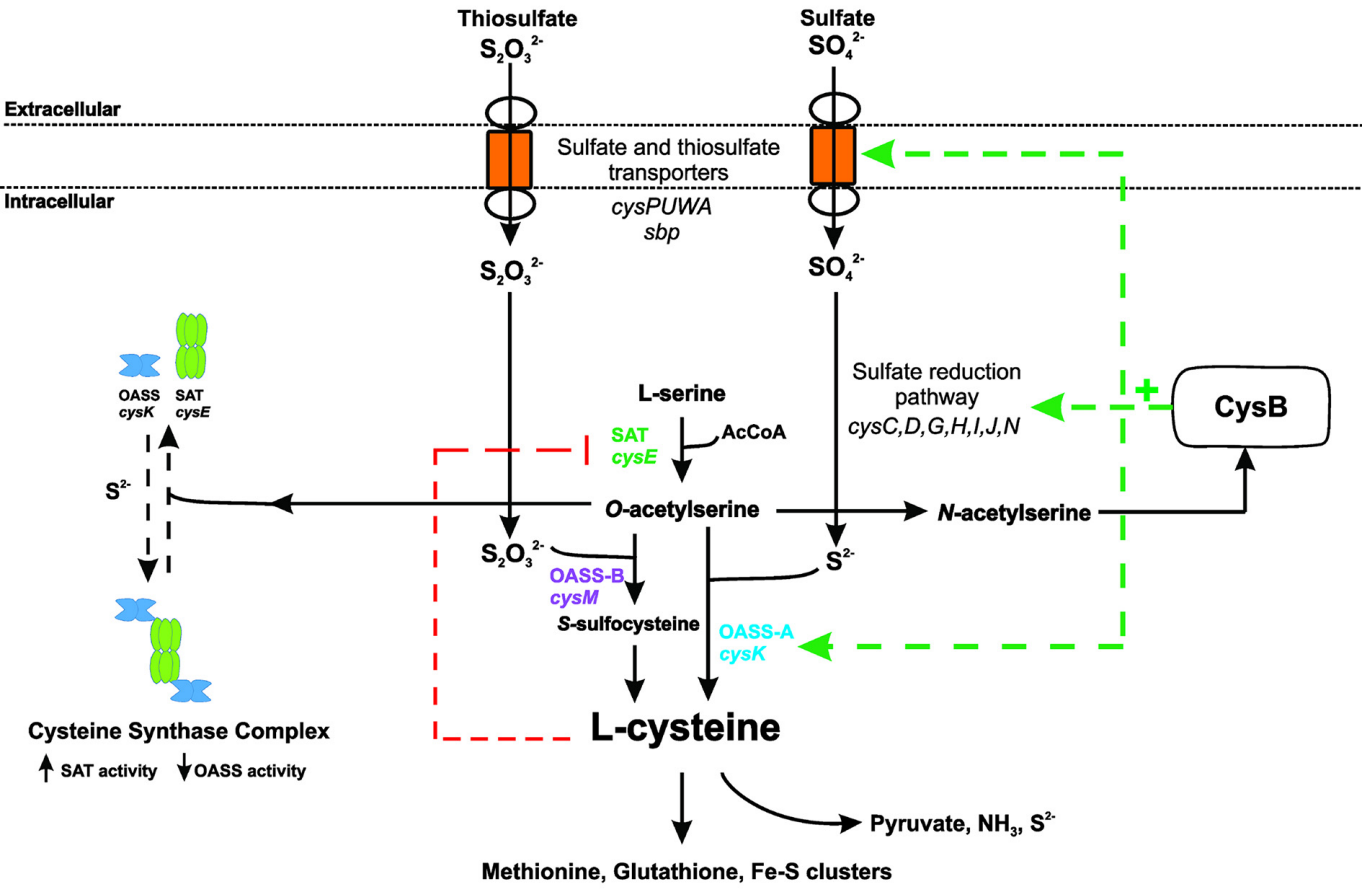
Overview of sulfur acquisition and assimilation pathways in bacteria Inorganic sulfur uptake and assimilation pathways converge to be separately condensed with *O*-acetylserine to form cysteine. Transcriptional regulator, CysB, up-regulates the sulfur uptake (*cysPUWA, sbp*) and sulfur reduction pathways (*cysC,D,G,H,I,J,N*), in the presence of the readily forming *O*-acetylserine isoform, *N*-acetylserine. Cysteine feeds into *de novo* synthesis of sulfur-containing metabolites, such as methionine, glutathione, thioredoxin and Fe-S clusters. Cysteine inhibits CysE via feedback inhibition. Cysteine can be catabolized by cysteine desulfhydrase to release pyruvate, ammonia and sulfide.

The synthesis of cysteine is a two-step process, catalyzed by two enzymes, serine acetyltransferase (SAT;CysE) catalyzes the first step, requiring L-serine and acetyl coenzyme A (acetyl-CoA) to produce *O*-acetylserine (OAS). *O-*acetylserine sulfhydrylase A (OASS-A, CysK) combines *O*-acetylserine with sulfide to produce cysteine, whereas the OASS-B (CysM) isoform can utilize both sulfide and thiosulfate as the sulfur source for the synthesis of cysteine. The reductive assimilation of sulfate to sulfide for the synthesis of cysteine is catalyzed by a suite of enzymes, often found in the sulfate reduction operon in bacteria (Figure 1). Thiosulfate is an alternative sulfur source used directly to synthesize cysteine by the OASS-B isoform, CysM (Figure 1). The enzymes required for sulfate assimilation are also being explored as potential antimicrobial targets but are outside the scope of this review.

The pathways of sulfur acquisition converge at cysteine (Figure 1). Therefore, the regulation of cysteine synthesis acts to control sulfur flux within the cell. Regulation occurs at both the genetic and protein levels. At the genetic level, the transcription factor CysB, belonging to the LysR family of transcriptional regulators, controls expression of key transporters of sulfur containing molecules, the sulfate reduction operon, and enzymes involved in cysteine synthesis, with the exception of CysE, which is not regulated by CysB (Figure 1) [14–18]. Whereas at the protein level, CysE which catalyzes the first step in the two-step reaction is inhibited by L-cysteine [19] and also forms a complex with CysK, termed the cysteine synthase complex (CSC) (Figure 1). While part of the CSC, CysE activity is enhanced [20], whereas CysK activity is inhibited due to the C-terminal peptide of CysE binding and occluding the active site of CysK [20]. Pathway intermediate *O*-acetylserine and its isomer *N*-acetylserine, along with sulfide, regulate formation of the CSC and act as inducers and anti-inducers of CysB, respectively.

In this review, we briefly summarize the structural and mechanistic features of the CysE and CysK/CysM enzymes from bacterial pathogens and the evidence that support targeting this pathway for the development of new antimicrobials. We provide a comparison of compounds identified thus far that inhibit the SAT and OASS enzymes and the methods used to identify these compounds.

## Serine acetyltransferase (CysE)

CysE is a serine acetyltransferase that catalyzes the first committed step of the cysteine biosynthetic pathway (Figure 1), utilizing L-serine and acetyl-CoA to synthesize the pathway intermediate *O*-acetylserine. Not only does CysE catalyze the first committed step, it is subject to feedback inhibition by the pathway end-product L-cysteine. CysE is nonessential in some bacterial pathogens, but curiously is essential in others, suggesting that CysE inhibition holds promise as a new antimicrobial target and/or as an antibacterial adjuvant.

## Essentiality and role of CysE during infection

CysE is important not only for the *de novo* synthesis of cysteine but also plays a key role in bacterial virulence. CysE is essential in the pathogens, *Staphylococcus aureus* [21], *Escherichia coli* O157:H7 strain [22], *Haemophilus influenzae* [23] and the pathogenic *Neisseria* species; *Neisseria gonorrhoeae* [24] and *Neisseria meningitidis* [25]. Essentiality of *cysE* in these bacteria was elucidated using transposon mutagenesis screens, and requires further validation. Interestingly, all screens were performed in culture media containing cysteine and other organic sulfur compounds, indicating that these bacteria have a requirement for the *de novo* synthesis of cysteine, despite the availability of extracellular organic sulfur sources. Furthermore, some of these organisms have non-functional sulfate assimilation pathways, such as *N. gonorrhoeae* [26] and *S. aureus* [27], precluding the reduction of sulfate to sulfide as a source of sulfur for cysteine synthesis. While these organisms can grow on the alternative sulfur sources, thiosulfate and sulfide [27,28], the essentiality of *cysE* in cysteine rich media suggests capability for the *de novo* synthesis of cysteine. It is also possible that CysE and/or the product *O*-acetylserine has an as yet unidentified function that makes it essential in these organisms.

In bacteria where CysE is nonessential and able to be deleted from the bacterial chromosome, growth defects and reduced virulence have been observed. For example, in the drug resistant pathogen *Klebsiella pneumoniae*, CysE is not essential, but when deleted the mutant exhibits decreased fitness in a mouse model of pneumonia, thereby playing an important role in lung infection [29]. Another key example is *M. tuberculosis*, where randomized transposon mutagenesis studies demonstrated profound effects for a number of sulfate reduction genes and *cysE* by gene disruption [30]. Further investigation demonstrated attenuation of *M. tuberculosis cysE* deletion strains in *in vitro* models of dormancy [31].

Furthermore, *cysE* deletion in the sheep pathogen *Brucella ovis* resulted in poor growth in rich media and an early entry into stationary phase [32]. *Brucella ovis ΔcysE* strains were more susceptible to oxidative stress, shown through increased sensitivity to hydrogen peroxide. Cell invasion assays revealed that deletion of *cysE* did not affect cell infection but did significantly reduce replication within macrophages [32]. While the deletion of *cysE* is non-lethal, it imposes a fitness cost on *B. ovis* during intracellular growth, demonstrating the requirement for cysteine biosynthesis for survival within the host.

Deletion of *cysE* can also influence bacterial antibiotic resistance. For example, an *E. coli* K12 *cysE* deletion strain had increased tolerance to the antibiotic novobiocin [33]. Conversely in the pathogen *S. typhimurium* loss of CysE function increased mecillinam sensitivity [34]. Supporting this increased antibiotic sensitivity phenotype, cysteine biosynthesis is crucial for swarm cell differentiation in *S. typhimurium*. Inactivation of cysteine biosynthetic genes resulting in cysteine auxotrophy led to increased antibiotic sensitivity in the swarm cell state [9], even though the swarm medium contained sufficient cysteine to support growth. There is a complex interplay between cysteine metabolism, oxidative stress and antibiotic resistance, under normal growth conditions. *Salmonella typhimurium* cysteine auxotrophs are oxidatively stressed and supporting this, in wild-type cells oxidative stress induces cysteine biosynthesis. *Salmonella typhimurium cysE* deletion strains are incapable of synthesizing cysteine and have decreased concentrations of reduced thiols or decreased total glutathione, leading to increased susceptibility to oxidative stress [10]. Differences seen between *E. coli* with increased tolerance to novobiocin and *S. typhimurium* could be due to the mechanism of action of the antibiotic or the presence/absence of cysteine/cystine or inorganic sulfur compounds in the culture media used in experiments.

CysE from *E. coli* and *Providencia stuartii* impacts biofilm formation in these pathogens [35]. Deletion of *cysE* from *E. coli* and *P. stuartii* enhanced biofilm formation. However, this could be reversed by supplementation with cysteine (100 μM) or *O-*acetylserine (10 mM) but not *N*-acetylserine [35]. The high concentration of *O-*acetylserine required for biofilm reduction compared to cysteine, suggests cysteine itself negatively regulates biofilm formation in a yet to be determined role. Given that bacteria in biofilms are less sensitive to antibiotics, inhibiting their formation could provide a novel way for enhancing current antibiotics.

## Structural characteristics of CysE

CysE (EC 2.3.1.30) belongs to the acetyltransferase family of hexapeptide acyltransferases. Members of this family are defined by a six-peptide tandem repeat, [LIV]-[GAED]-X_2_-[STAV]-X, which gives rise to a distinctive left-handed beta helix (LβH) [36]. Structural characterization of CysE enzymes with and without substrates and cysteine (inhibitor) bound from a range of Gram-negative bacterial pathogens including *E. coli* (1T3D) [37], *H. influenzae* (1SSM, 1SSQ, 1SST) [38], *Yersinia pestis* (3GVD) [39], *Brucella melitensis* (3MC4), *Vibrio cholerae* (4H7O), *Brucella abortus* (4HZC, 4HZD) [40], *K. pneumoniae* (6JVU) [41], *N. gonorrhoeae* (6WYE, 7RA4) [42] and *S. typhimurium* (7E3Y). These structures provide insight into active site architecture upon substrate and inhibitor binding, which can be used to inform inhibitor design.

**Figure 2:**
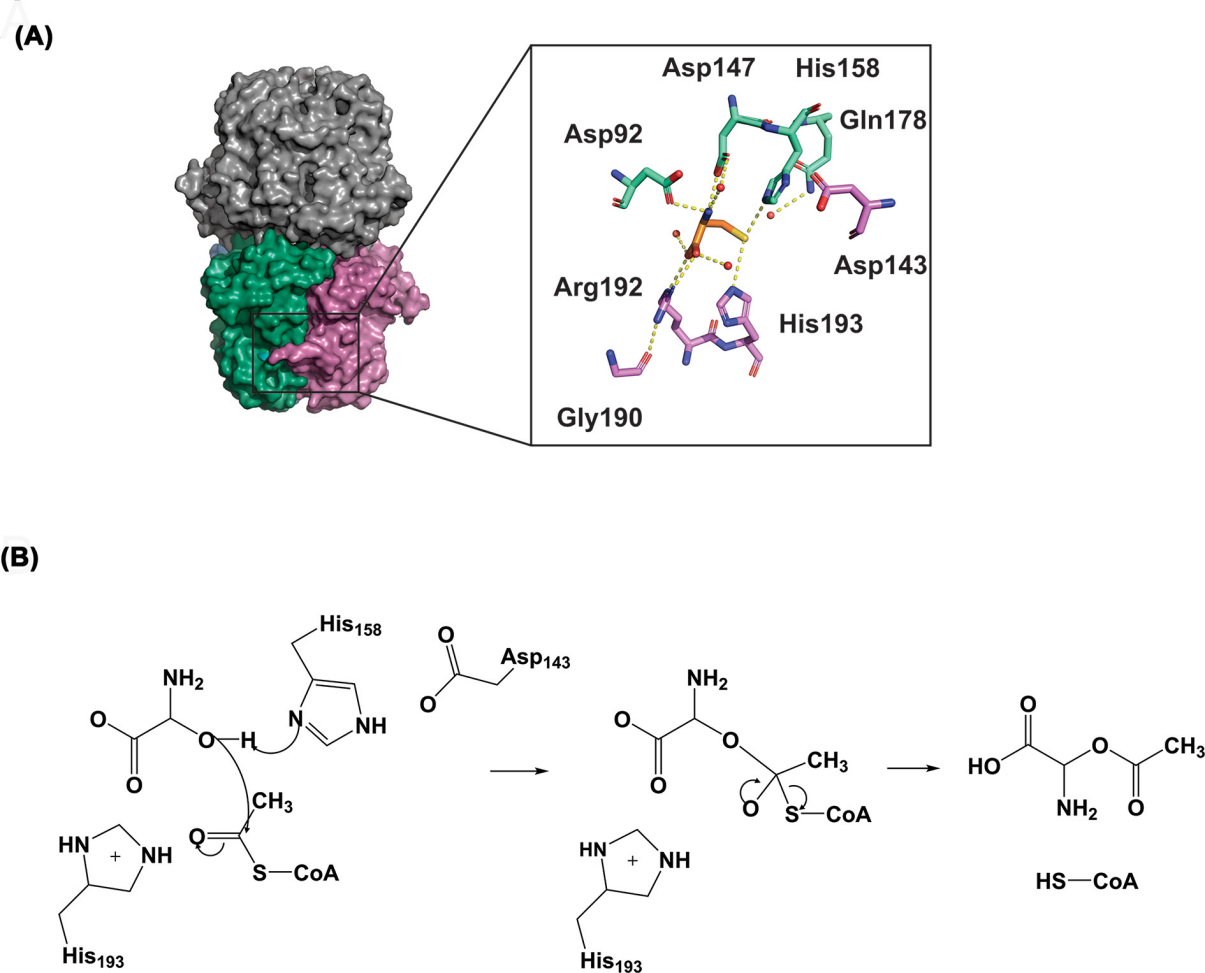
L-cysteine interactions with L-serine binding pocket in CysE from *E. coli* (1T3D) (**A**) Active site residues are represented as sticks, colored green (Asp92, Asp147, His158 and Gln178) and pink (Asp143, Gly190, Arg192 and His193), based on chains. Inhibitor L-cysteine is represented as orange sticks. Hydrogen bonds are shown as yellow dashed lines. (**B**) CysE reaction mechanism for formation of *O*-acetylserine, adapted from [37]. Figure was produced using PyMOL and ChemDraw Prime (RRID:SCR_016768).

The CysE monomer consists of an amino-terminus (N-terminus) α-helical domain and a carboxy terminus (C-terminus) LβH domain (Figure 2A). The monomers assemble to form a trimer, which in turn forms a functional hexamer through hydrophobic trimer–trimer interactions via the alpha helices of the N-terminal domains (Figure 2A) [37,38,42,43]. There are six active sites in the hexamer, formed between adjacent monomers of the C-terminal LβH domain. There is one deviation from the hexapeptide repeat, producing a meandering loop which forms part of the active site [37].

The hexameric structure of CysE enzymes differs from other members of the acetyltransferase family, which are active trimers. The CysE hexamer forms the cysteine synthase complex (CSC) with the OASS-A/CysK enzyme. There are exceptions to this hexameric configuration, such as the CysE isoforms from the protozoan parasite *Entamoeba histolytica*, where CysE is an active trimer [44].

## CysE enzyme mechanism

Inhibitor design also relies on a detailed understanding of the enzyme’s kinetic mechanism as well as structural features. CysE catalyzes the acetyl-CoA dependent acetylation of the hydroxyl side chain of L-serine to form *O*-acetylserine. CysE represents a key regulatory mechanism for the cysteine biosynthetic pathway due to its feedback inhibition by L-cysteine [19,38]. CysE is constitutively expressed and is regulated post-translationally through formation of the CSC [37]. Kinetic characterization of CysE enzymes shows a ternary mechanism, with a random order mechanism reported [45]. During catalysis, a conserved catalytic histidine (His158; *E. coli* CysE numbering) acting as a general base attacks the hydroxyl side chain of L-serine, which is stabilized by a neighboring aspartate (Asp143; *E. coli* CysE numbering), forming an intermediate, allowing for transfer of the acetyl group from acetyl-CoA to L-serine, releasing products *O-*acetylserine and coenzyme A [37] (Figure 2). While there is sequence divergence amongst CysE homologues, there is strong conservation of mechanism and active site residues [37].

## Cysteine inhibition of CysE

Tight control of intracellular cysteine levels is essential for meeting the cysteine requirements of the cell, while preventing unwanted toxic effects of high cysteine concentrations [46]. As mentioned previously, CysE is constitutively expressed [47], where the main form of regulation is through formation of the CSC and feedback inhibition by the pathway product L-cysteine [19]. L-cysteine is a potent inhibitor of bacterial CysE enzymes with IC_50_ values of 0.5–10 µM reported [19,20,42,45]. Kinetic studies show that cysteine is a competitive inhibitor relative to serine, through binding to the serine binding pocket which has been confirmed through crystallography [37,38,40] (Figure 2). Interestingly, cysteine displays competitive inhibition relative to acetyl-CoA even though it binds to the serine binding pocket [42,48,49]. This competitive inhibition is explained by observing CysE crystal structures with L-cystein bound. Upon binding of L-cysteine in the serine binding site the C-terminal tail folds up against the CysE monomer, physically blocking the active site and preventing the binding of acetyl-CoA [38]. Supporting this, truncation of the last ten C-terminal residues that form the C-terminal tail reduces CysE sensitivity to cysteine inhibition [50]. This is thought to prevent the accidental acetylation of L-cysteine, given its structural similarity to serine. [50]

## Development of CysE inhibitors

CysE enzymes from bacterial pathogens have been extensively characterized, both kinetically and structurally, with numerous high-quality crystal structurers available for inhibitor design. Given the importance of CysE in infection and antibiotic resistance, and its essentiality in some bacterial pathogens, CysE represents an attractive drug target. The inhibition of CysE would deplete the cell of cysteine and *O*-acetylserine, where the later isomerizes to *N*-acetylserine, the natural inducer of the cysteine biosynthetic operon, leading to metabolic dysregulation. There have been limited studies into inhibitors of CysE enzymes, but promising inhibitors (IC_50_ ≤ 100 µM) have been identified for a number of bacterial pathogens (Table 1) and are discussed below.

**Table 1:** List of characterized CysE inhibitors

Inhibitor	Enzyme	IC_50_ (µM)^*^	*K*_i_ (µM) AcCoA^*^	*K*_i_ (µM) L-serine	Citation
Compound 3	*Ec*CysE	72^†^	42†	ND	[54]
Compound 4	*Sa*CysE	71.84 ± 0.27	225.3^†^	53.9†	[53]
Compound 30		71.84 ± 0.15	111.5^†^	47.66^†^	[53]
Quercetin	*Kp*CysE	3.7†	162	ND	[41]
Compound 3a	*St*CysE	48.6 ± 8.43	ND	ND	[56]
Compound 5		110 ± 0	64 ± 12	ND	[55]
Compound 22d		4.24 ± 0.11	ND	ND	[55]

ND, Not determined.

* Error reported as standard error.

† No error reported.

## Natural compound inhibitors

**Figure 3:**
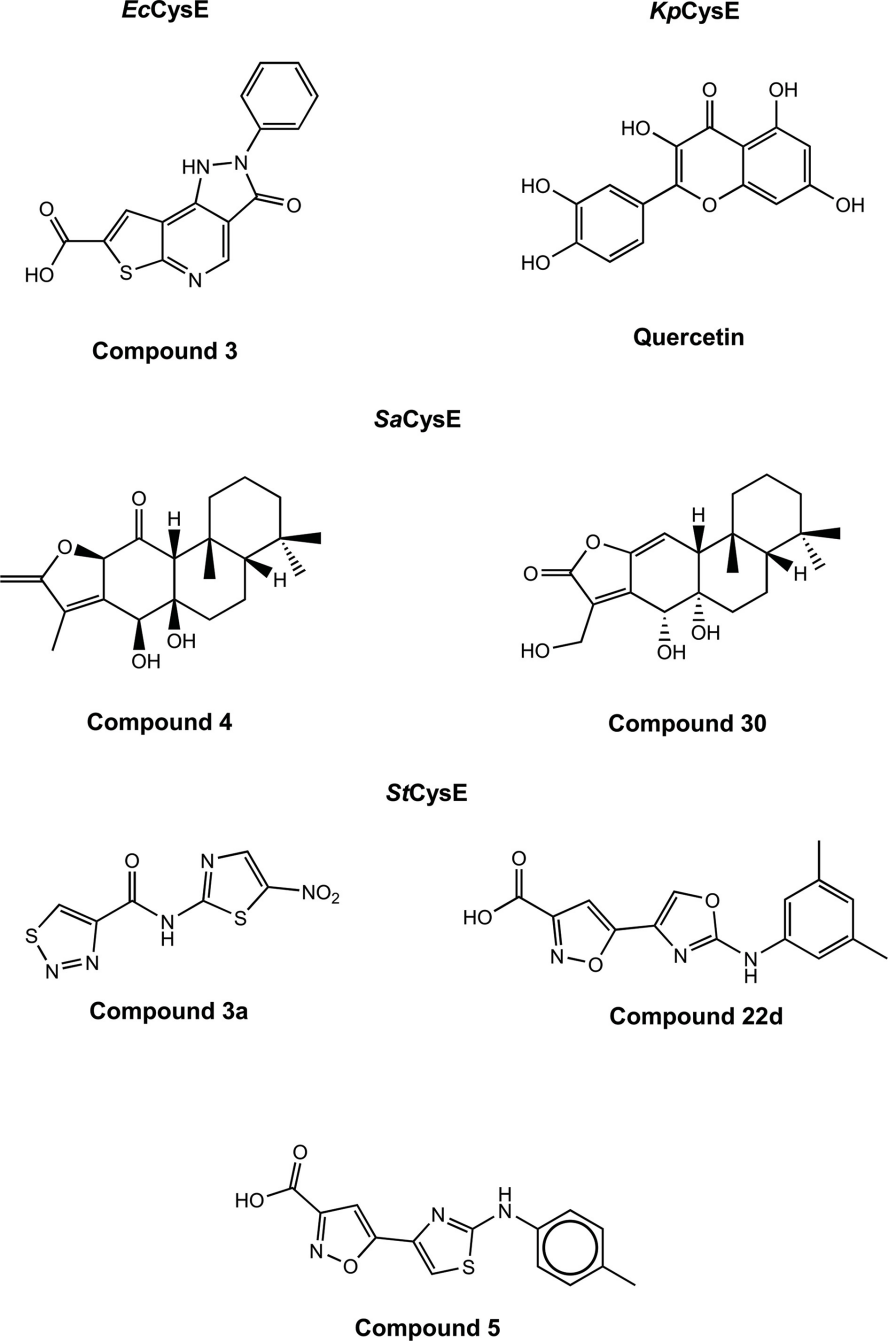
Chemical structures of CysE inhibitors Figure produced using ChemDraw Prime (RRID:SCR_016768).

Several promising CysE inhibitors have been identified via *in silico* screening of natural compound libraries. Recently, the flavonoid quercetin was found to inhibit CysE from *K. pneumoniae* (IC_50_ = 3.7 μM) (Table 1) [41]. Through docking analysis, quercetin (Figure 3) was shown to bind allosterically to the CysE trimer–trimer interface. Although not experimentally investigated by the authors, the binding of quercetin to this interface may inhibit *Kp*CysE through disrupting the trimer–trimer interactions, dissociating the hexamer, which has been shown to reduce CysE activity [41]. However, quercetin has been shown to inhibit other bacterial enzymes including isocitrate lyase [51] and glutamate racemase [52]. This broad inhibition suggests that quercetin inhibition of CysE might be non-specific, which is supported by the targeting of the trimeric interface and not serine or acetyl-CoA binding sites.

Natural compound inhibitors have also been identified for *Sa*CysE from methicillin-resistant *S. aureus* (MRSA) [53]. These include two polycyclic diterpenoids; compound 4 (11-oxo-ebracteolatanolide B) and compound 30 ((4R,4aR)-dihydroxy-3-hydroxymethyl-7,7,10a-trimethyl-2,4,4a,5,6,6a,7,8,9,10,10a,l0b-dodecahydrophenanthro[3,2-b]furan-2-one) (Figure 3). These compounds share the same chemical scaffold, with substitution of oxygens attached to the phenyl rings. Both compounds inhibited *Sa*CysE (both IC_50_ = 71.84 µM), where compound 4 was shown to display mixed inhibition against serine and competitive inhibition against acetyl-CoA, while the opposite was seen for compound 30. Docking analysis with a structural homology model of *Sa*CysE shows hydrophilic interactions between compound 4 and key catalytic residue, His95, and the N-terminal domain residues, Ala43 and Gly44. Compound 30 interacts with these identical residues and active site residue Asp94. Given the highly similar chemical structure of these compounds, it is unsurprising that they share CysE residue interactions; however what is interesting is these compounds do not dock in either the acetyl-CoA or serine binding pocket, but instead bind in a pocket between the third α-helix (equivalent to sixth α-helix in *E. coli*, 1T3D) and the serine binding site. This is a unique method of targeting the CysE active site as the inhibitor can interact with the active site residues without occupying the active site, and may explain the mixed and competitive inhibition observed for these compounds.

Both compounds were also able to inhibit MRSA growth with minimum inhibitory concentration (MIC) values of 12.5 and 25 µg/ml for compound 4 and 30, respectively [53]. Furthermore, both compounds were able to disrupt a mature MRSA-biofilm at one-fold the MIC concentration, and did not display any cytotoxicity to human cells. Both compounds did not inhibit the structurally similar hexapeptide enzyme, GlmU (N-acetylglucosamine-1-phosphate uridyltransferase) demonstrating target specificity. Although the *in vitro* IC_50_ values are relatively high, given that these compounds inhibited growth of the target organism, displayed target specificity and are well tolerated by mammalian cells, these compounds are ideal for further optimization. Natural compounds have become popular for identifying novel antimicrobials, a large number of chemically distinct compounds can be screened and optimized by substituting chemical groups. Promising natural compound inhibitors discussed here could also be used for targeting other CysE homologs.

## Chemical inhibitors

Initial research into the development of CysE inhibitors was by conducted by Agarwal et al. (2008) [54]. The researchers employed virtual screening to identify inhibitors of CysE from *E. coli*. Screening of the crystal structure of *Ec*CysE (1T3D), identified nine compounds with promising docking scores, of which three were characterized *in vitro*. Compound 3, (3-oxo-2-phenyl-3,5-dihydro-2H-pyrazolo(3,4d)thieno(2,3-b)pyridine-7-carboxylic acid), was the only compound identified to inhibit *Ec*CysE (72 µM) and exhibit antimicrobial effects. This compound was tested for growth inhibition of the parasite *E. histolytica* resulting in unexpectedly potent inhibition (IC_50_ = 0.61 µM), suggesting off-target inhibition.

As well as natural products, 2-aminothiazole and 2-aminooxazole compounds have been investigated as inhibitors of CysE enzymes. These compounds mimic binding interactions with key active site residues similar to the natural inhibitor L-cysteine (Figure 2). Recent studies have explored 2-aminothiazoles and 2-aminooxazole compounds as inhibitors of CysE from *S. typhimurium* (*St*CysE) [55,56]. Since the crystal structure for *St*CysE (7E3Y) has been solved only recently [57], all virtual screening was carried out against both *Ec*CysE (1T3D) and *H. influenzae* CysE (1SSM) crystal structures, as there is strong conservation of active site residues with *St*CysE. Virtual screening of ∼91,000 compounds from three libraries identified six compounds, which *in vitro* had IC_50_ values ranging from 13.6 to 84.1 μM [56]. Further characterization of these compounds, revealed only compound 3a (N-(5-Nitro-1,3-thiazol-2-yl)-1,2,3-thiadiazole-4-carboxamide), a 2-aminothiazole (Figure 3), to inhibit *St*CysE with an IC_50_ of 48.6 μM to be bactericidal, with an MIC of 64 µg/mL against *E. coli*. *In silico* docking analysis showed that compound 3a interacts with key active site residues Asp92, Asp157, Arg192, His193 and catalytic His158, mimicking interactions exhibited by the inhibitor L-cysteine (Figure 2). Compound 3a was shown to inhibit *E. coli* growth, but only in media low in cysteine. Previous research supports the anti-bacterial activity of 2-aminothiazoles, with bactericidal activity against *M. tuberculosis* reported [58,59].

Further research into *St*CysE inhibition was conducted using an in-house library for further virtual screening of *St*CysE [55,57]. Using the same screening method as discussed previously the researchers identified seven compounds that reduced *St*CysE activity, with the most potent being the substituted 2-aminothiazole, compound 5, (5-{2-[(4-Methylphenyl)amino]-1,3-thiazol-4-yl}-1,2-oxazole-3-carboxylic acid) (Figure 3), with an IC_50_ of 110 µM, which displayed competitive inhibition relative to acetyl-CoA (K_i_ = 64 μM). Docking analysis showed compound 5 interacts with the CysE serine active site residues and acetyl-CoA binding pocket, where the carboxylic acid group interacts with the same residues as seen for inhibitor cysteine/substrate serine. The ‘L-shape’ of the inhibitor allows it to mimic acetyl-CoA, explaining the observation of competitive inhibition [55]. Structure–activity relationship analysis was undertaken through *in vitro* screening of compound 5 analogues. Substitution of the 2-aminothiazole ring with a 2-aminooxazole was shown to increase affinity, and the presence of an ester, amide or carboxylic acid group connected to the isoxazole ring was shown to be essential for affinity [55]. Isoxazole-3-ester and isoxazole-3-carboxylic acid derivatives were further optimized through synthesis with different chemical groups connected to the oxazole ring [55]. Affinity was not substantially affected by the side group, but derivatives with electron-withdrawing groups were unstable compared with electron-donating groups. The most potent analogue was compound 22d, (3,5-dimethylphenyl-(2-aminooxazol-4-yl) isoxazole-3- carboxylic Acid) (Figure 3), with an IC_50_ of 4.2 µM (Table 1). Unfortunately, this compound was unable to inhibit the growth of *E. coli*, requiring further optimization to improve compound permeability.

Overall, CysE inhibitor development is in its early stages, with a number of different strategies employed, with the main challenge in obtaining potent inhibitors that also inhibit bacterial growth. The essentiality of CysE in the notoriously antibiotic-resistant pathogens *S. aureus* and *N. gonorrhoeae* highlights CysE as an ideal target for antimicrobial development. With more research being undertaken in targeting cysteine biosynthetic enzymes, overcoming the challenge of finding compounds that are potent and yet specific, while being permeable to target organisms, will lead to the development of promising inihibitors.

## *O*-acetylserine sulfhydrylase (CysK/CysM)

*O*-acetylserine sulfhydrylase is a pyridoxal 5′phosphate (PLP) dependent enzyme that catalyzes the second step of the L-cysteine biosynthesis reaction, combining *O-*acetylserine and a sulfur donor into cysteine. OASS is present in bacteria as two isoforms, OASS-A (CysK) that utilizes sulfide for the synthesis of cysteine, and OASS-B (CysM) that utilizes sulfide and thiosulfate. Bacteria with *cysK* or *cysM* deleted from the genome exhibit reduced virulence, compromised fitness and decreased antibiotic resistance. Subsequently, its inactivation is being pursued as a strategy for the identification of novel antibiotics and/or antibiotic adjuvants that target non-essential proteins.

## Role and essentiality of CysK/CysM in bacterial pathogens

As the second and final enzyme in the cysteine biosynthetic pathway both OASS isoforms play an important role in bacteria. Transposon mutagenesis screening found that CysK is essential in just two bacterial pathogens, *Campylobacter jejuni* [60], and *Francisella novicida* [61]. Like CysK, CysM is essential in very few bacterial species, including *Burkholderia pseudomallei* [62], and two strains of *Burkholderia cenocepacia*, K56-2 [63], and J2315 [64]. Given that many bacterial species have both OASS isoforms, or even two copies of CysK, it is not surprising CysK and/or CysM are non-essential in many bacterial pathogens. For example, the *M. tuberculosis* genome contains three annotated OASS genes, denoted CysK1, CysK2 and CysM. The nomenclature of these genes is confusing in that OASS-A is denoted as CysK1, OASS-B as CysK2 (not CysM) and the mycobacterial CysM is unique and found only in actinobacteria. *Mtb*CysM uses a small thiocarboxylated protein (CysO) as the sulfur donor and *O*-phosphoserine (not *O*-acetylserine) as the preferred acceptor substrate [12,65–67]. Disrupting the *de novo* cysteine biosynthesis pathway in *M. tuberculosi*s represents an attractive drug target. Cysteine biosynthesis is consistently upregulated in dormancy models of infection [68,69], particularly survival of *M. tuberculosis* in infected macrophages in the granuloma, where it is exposed to an extremely hostile environment. It could be argued that *M. tuberculosis* could obtain cysteine from the host and not be dependent on *de novo* synthesis of cysteine. However, the up-regulation of sulfur acquisition and cysteine synthesis genes in persister cells suggests that the host does not provide a sufficient amount of cysteine [68–72], and it is likely cysteine is scarce within the granuloma due to host defense strategies such as nutrient depletion. *Mycobacterium tuberculosis cysO* and *cysM* deletion strains show attenuation in *in vitro* models of dormancy and also in a mouse model of infection [31]. A target identification pipeline for drug targets in *M. tuberculosis* based on a comprehensive *in silico* analysis using experimental derived phenotype data and proteomics, suggests that both CysE and CysK2 are high confidence drug targets [73].

As discussed earlier, inactivation of the cysteine biosynthetic operon leading to cysteine auxotrophy in *S. typhimurium* led to an increased susceptibility to antibiotics during swarming, which is normally associated with a decreased susceptibility to antibiotics. A *S. typhimurium Δcysk, cysM* double deletion strain was pleiotropic [10], making it difficult to associate a particular phenotype to this strain, presumably due to the accumulation of toxic intermediates, such as 3′phosphoadenoside 5′-phosphosulfate [74].

Shatalin et al. (2011) linked both the high concentration of H_2_S, and decreased cysteine concentration to increased resistance to a variety of antibiotics. H_2_S is cytoprotective in some bacteria due to its ability to suppress oxidative stress generated by some antibiotics [75]. An *S. typhimurium cysK* deletion strain had decreased cysteine production, resulting in H_2_S accumulation causing an eight-fold higher resistance to ofloxacin compared to wild-type [76]. This highlights the various roles of CysK in antibiotic susceptibility, enhancing resistance to some and decreasing resistance to others, due to the target of the antibiotic and degree of oxidative stress within the cell.

Metal ions at low concentrations are beneficial to bacteria, however, can become toxic at higher concentrations causing oxidative stress and eventually cell death [77]. Studies in *S. typhimurium* LT2 and *E. coli* demonstrated that CysK plays an essential role in mediating resistance to the metal ion, tellurite (K_2_TeO_3_) [78,79], which exhibits strong oxidizing properties through an unknown mechanism. Deletion of *cysK* from *Azospirllum brasilense* conferred a lower MIC when grown on media with tellurite, whilst transfer of the *A. brasilense* and *Bacillus stearothermophilus cysK* into *E. coli* and *S. typhimurium* respectively, conferred increased tellurite resistance [78,79].

As mentioned previously, disruption of the cysteine biosynthetic pathways can affect biofilm formation. The effect of a number of mutants from the cysteine biosynthetic operon of *Vibrio fischeri*, including Δ*cysH*, Δ*cysJ*, Δ*cysK* and Δ*cysN* on biofilm formation were tested, with the greatest effect on biofilm formation seen with the Δ*cysK* mutant [80]. Biofilm and pellicle formation are vital to colonization which was observed in early colonization of baby squid where Δ*cysK* resulted in decreased colonization [80]. Addition of cysteine allowed rescue of the biofilm defect and partial rescue of the pellicle defect, indicating a key role of CysK in initial colonization [80].

In a screen for genes important for the switch of *N. meningitidis* from commensal to pathogen, CysK was identified as being important in this switch. Saturated random transposon insertion libraries of *N. meningitidis* were engineered and assessed for fitness during normal growth and colonization of epithelial and endothelial cells, and the CysK mutant was identified as being of particular importance for epithelial cell infection [81].

## Structural characteristics of CysK and CysM

*O-*Acetylserine sulfhydrylase (OASS; EC 2.5.1.47) belongs to the tryptophan synthase β-superfamily, and the β-family of PLP dependent enzymes [15,82,83]. PLP is an essential cofactor, utilized in the active site of OASS enzymes. As briefly discussed above there are two OASS isoforms, which each use an alternate sulfur source; CysK utilizes sulfide, whereas, CysM utilizes both thiosulfate and sulfide, with *O*-acetylserine to form cysteine [84]. Both isoforms are present in most bacteria, enabling the utilization of both thiosulfate and sulfide for cysteine biosynthesis. Expression of these isoforms is influenced by aerobic or anaerobic growth, for example, the genome of the pathogen *S. typhimurium* encodes both OASS isoforms with CysK expressed in excess of CysM under aerobic conditions, and vice versa under anaerobic conditions [85]. The two isozymes, CysM and CysK, function as homodimers and exhibit 25–45% similarity in their amino acid sequence [66,86–88]. The key difference between these isoenzymes is the ability of CysM to utilize larger sulfur donor substrates including thiosulfate, where CysK is only capable of accepting the small sulfur donor, hydrogen sulfide [67,87,89].

CysK and CysM enzymes have been structurally characterized from a range of a bacterial species, enabling comparison of the two isoforms, and providing insight into active site conformation and isoform differences for isoform specific inhibitor design. Within the CysK and CysM homodimer one PLP molecule is bound per subunit in the active site cleft that is formed between the N and C-terminal domains of each monomer. The active site cleft of CysK and CysM are fairly similar and are lined by seven chain segments totaling 16 residues. Six of these chain segments are highly conserved between CysK and CysM isoforms, however, the seventh segment spanning residues 210-216 of CysM (*E. coli* CysM numbering) indicates a key difference between the two enzymes [67,90]. CysM contains the three large residues Arg210, Arg211 and Trp212 followed by a three-residue insertion, which bulges toward the surface enlarging the active site. Most of this enlarged active site is occupied by the sidechain tail of Arg210 which most likely binds to thiosulfate or other larger sulfur donor substrates [67]. Conversely, *St*CysK has three small residues, Gly230, Ala231 and Gly232 [67,91]. This small change reduces the size of the active site cleft, therefore restricting CysK to using bisulfide as its sulfur donor. Lys41, is highly conserved across both CysK and CysM isozymes as it forms an internal aldimine linkage with PLP in both isoforms [91,92]. The enzyme cycles through open and closed conformations during catalysis. Both open (no substrates bound) and closed forms (substrates bound) have been structurally characterized [90,93,94] with the closed form occurring via binding of the substrate α-carboxylate or acetate to the substrate binding loop (residues 68–71 in *S. typhimurium*) triggering the active site to close [90,94].

The two monomer subunits interact solely at the dimer interface and an allosteric binding site was recently identified in the CysK isoform, that is not present in CysM [91]. The structure of CysK with Cl^−^ bound at the allosteric anion binding site at the subunit interface, shows a closed/inhibited form of the enzyme [95]. The anion sulfide behaves similarly to chloride and has essentially the same ionic radii and also binds to the anion binding site [95], presumably acting as a further regulator of CysK activity and modulating sulfur flux within the cell.

## CysK/CysM enzyme mechanism

**Figure 4:**
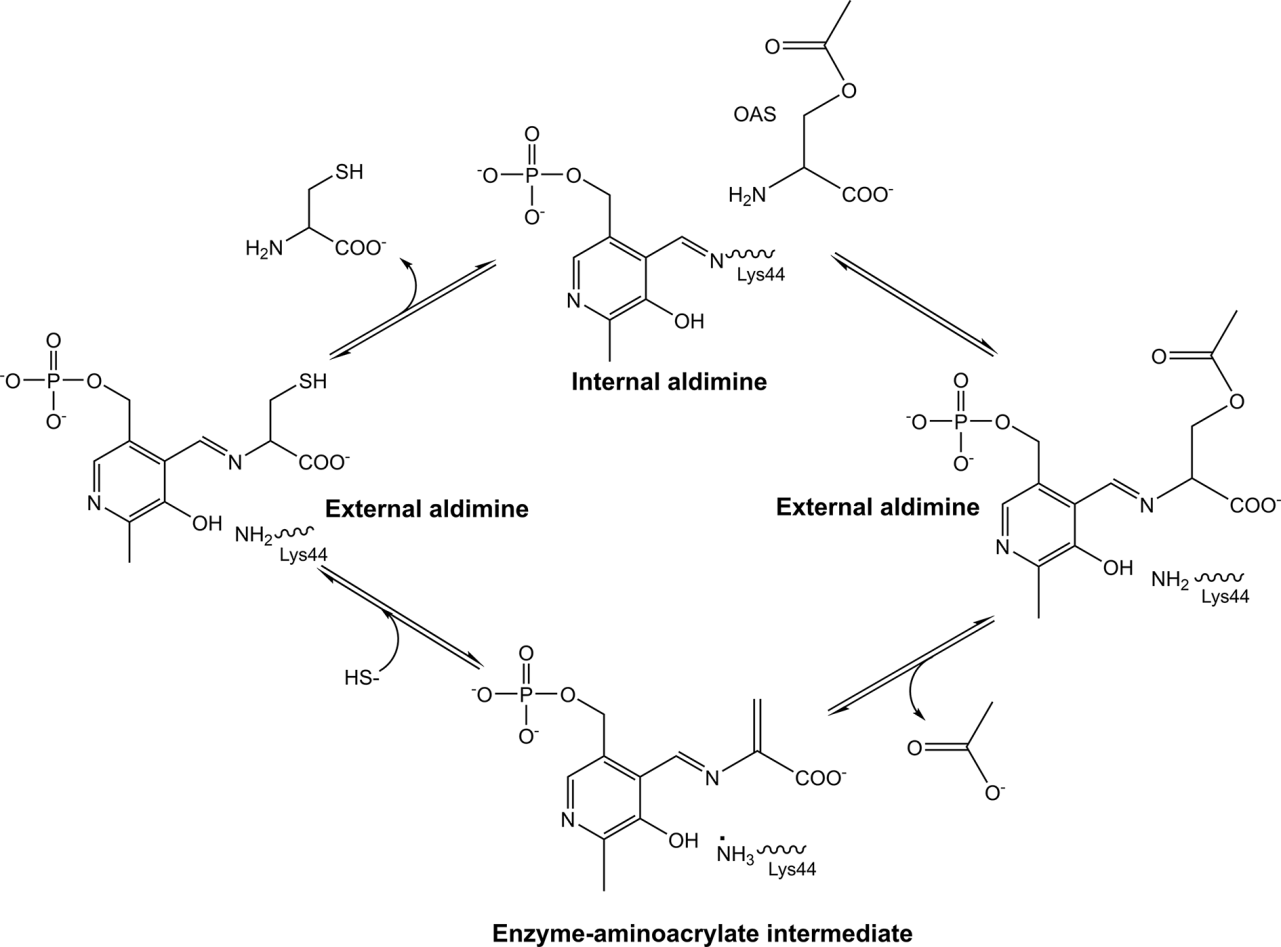
CysK catalytic mechanism, based on the *M. tuberculosis* CysK enzyme (*Mtb*CysK Lys44 equivalent to Lys41 in text) Figure produced using ChemDraw Prime (RRID:SCR_016768), adapted from [77].

As discussed for CysE, a detailed understanding of both structure and enzyme mechanism is important for inhibitor design. OASS catalyzes the replacement of the β-acetoxy group of *O*-acetylserine by either sulfide in the case of CysK or thiosulfate and sulfide in the case of CysM to produce L-cysteine. Both CysM and CysK utilize a bi-bi ping-pong mechanism for cysteine biosynthesis [87,88]. This involves the release of one of the half reactions products before all substrates have bound to the enzyme, generating an enzyme intermediate in the process [96]. *O*-Acetylserine carries out nucleophilic attack on the C-4′ of the internal aldimine (Figure 4). As the external aldimine is formed, the active site closes due to interaction of the substrate-carboxylate with an asparagine loop (Figure 4). A conserved lysine (Lys41, *E. coli* numbering), initially part of the internal aldimine PLP linkage, serves as a general base to deprotonate Cα in the β-elimination reaction that releases acetate at the conclusion of the first half reaction forming the α-aminoacrylate intermediate (Figure 4) [77,91]. Acetate diffuses out of the active site as it opens partially to allow entry of bisulfide and product release. Lys41 remains protonated at the beginning of the second half reaction, bisulfide diffuses into the active site attacking the Cβ of the α-aminoacrylate giving the cysteine external Schiff base (Figure 4). The cysteine product, *S-*sulfocysteine for CysM, and L-cysteine for CysK, is expelled via transimination [77,91]. *S-*sulfocysteine is reduced to L-cysteine via an unknown mechanism [84].

## Cysteine synthase complex formation

The bienzyme cysteine synthase complex (CSC) was first discovered during the purification of *S. typhimurium* CysK and CysE [19,97]. CSC formation has since been confirmed across many other species including *E. coli* [20,50,98], *H. influenzae* [99,100] and various plant species [101,102]. The formation of the CSC represents a unique avenue for the design of inhibitors. The complex forms via binding of the CysE C-terminal tail into the CysK active site, thus inhibiting CysK activity and therefore cysteine production [6,20,50,98–101]. The complex forms in 3:2 CysE:CysK ratio consisting of one CysE hexamer and two CysK dimers [99]. Deletion of 20 C-terminal residues from CysE results in an inability to bind to CysK and formation of the CSC does not occur, highlighting the importance of the C-terminal peptide tail of CysE in CSC formation [50]. CSC formation reduces L-cysteine feedback inhibition and L-serine substrate inhibition of CysE activity in *E. coli* [20]. Complex formation also reduces CysE cold inactivation at both 0 and 10°C [98], presumably due to increased stability caused by structural reorganization at part of the N-terminal domain of CysE, that interacts with CysK leading to allosteric stabilization at the interface between the CysE trimers [103]. CysM and CysE have no interactions and do not form a complex, due to differences in the active site structure [88].

Structural studies of CysK in complex with CysE C-terminal peptides have provided insight into the interaction of the C-terminal CysE tail with the active site of CysK as to date there are no atomic structures of the CSC. The PLP cofactor in the OASS active site has fluorescent properties sensitive to its microenvironment and protein conformational changes. These fluorescent properties can be used to monitor formation of the CSC with CysE and with peptides that mimic the C-terminal tail of CysE [104]. Fluorescent monitoring of the PLP cofactor binding to the entire CysE protein and a C-terminal decapeptide (mimicking the CysE C-terminal tail), demonstrated that the C-terminal α-carboxylate of the CysE C-terminal decapeptide and the CysE C-terminal tail fit into the same position [94,104].

At a ratio of 5:1 CysE:CysK (at which full complex formation is assumed to have taken place) the activity of CysK in the *E. coli* CSC is reduced to 10% of free CysK activity [50,98,105]. Yet the CysE C-terminal decapeptide when bound to CysK reduced activity to 50% at a 500:1 molar ratio of decapeptide to CysK [50]. This can be attributed to full length CysE binding 250-fold tighter to CysK compared with the C-terminal decapeptide [99]. Dissociation constants (*K*_D_) of peptides in complex with CysK compared with full-length CysE in complex with CysK further show the stark contrast in binding affinity of peptides compared with the full-length CysE in the CSC (Table 2). This indicates there may be additional structural features of CysE that CysK recognizes aside from the C-terminal decapeptide, which increases the binding affinity but are not sufficient for directing complex formation without the presence of the C-terminal decapeptide [99].

**Table 2:** Dissociation constants (*K*_D_) of CysE decapeptides and full-length CysE in the CSC

	CysE Decapeptide		Full-length CysE		
	HiDK	HiDStK	EcCSC1	EcCSC2	StCSC
K_D_ (nM)	515 ± 29^*^	972 ± 62^*^	4.5/6†	0.63^†^	0.83†

H*i*DK = *Hi*CysE C-terminal decapeptide (GIDDGMNLNI) with *Hi*CysK from [104]. *Hi*DStK = *Hi*CysE C-terminal decapeptide (GIDDGMNLNI) with *St*CysK from [104]. *Ec*CS C1 = full-length *Ec*CysE with *Ec*CysK from [20]. *Ec*CS C2 = full-length *Ec*CysE with *Ec*CysK from [106]. *St*CSC C = full-length *St*CysE with *St*CysK from [106].

* Error reported as standard error.

†No error reported.

**Figure 5:**
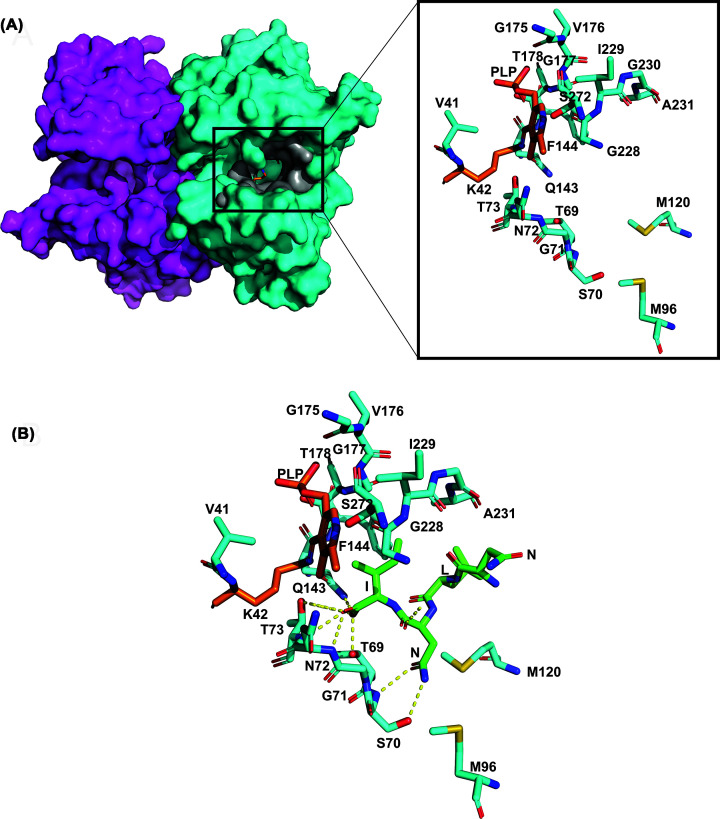
Overview of *H. influenzae* CysK (**A**) *Hi*CysK dimer surface shown with one monomer in magenta and the other in cyan with PLP bound to K42 visible in orange deep in the active site cleft. Zoomed into *H. influenzae* active site residues (V41, K42, 69-TSGNT-73, M96, M120, 143-QF-144, 175-GVGT-178, 228-GIGA-231 and S272) shown in cyan with PLP bound to K42 in orange. Active site is similar to *St*CysK open (0.5 Å, r.m.s.d) and *St*CysK partially closed anion-inhibited (0.4 Å, r.m.s.d) conformation [99] (**B**) *Haemophilus influenzae* active site residues shown in cyan interacting with bound *Hi*CysE C-terminal tetrapeptide shown in green (NLNI). Polar bonds shown with dotted yellow lines. Figure created with 1Y7L from [99] using PyMOL.

The highly conserved C-terminal isoleucine of the CysE C-terminal peptide is an essential anchor point for correct positioning of the C-terminal tail; in *H. influenzae* accounting for 80% of the total interaction energy [100]. This energy contribution is derived from Thr69 and Thr73 hydrogen bonds to the Ile267 α-carboxylate (Figure 5) [100]. The Ile267 sidechain forms hydrophobic interactions in an apolar pocket formed by the Phe144 and PLP (Figure 5) [100]. A further 10% of the total interaction energy is split equally between the C-terminal Asn266 and Leu265 (Figure 5) [100].

The dependence of the CSC on sulfur availability indicates regulation of sulfur flux to be the purpose of CSC formation [20,97,107,108]. The regulation forms a loop beginning with high availability of sulfur to the cell where the CSC is stabilized by bisulfide [97]; however, when sulfur availability is low, OAS accumulates via CysE production, thus indicating sulfur starvation and dissociation of the CSC [20,97,107,108]. Dissociation of the CSC can occur at OAS concentrations upward of 50 µM [97,107], which then non-enzymatically isomerizes to *N*-acetylserine and binds to the transcriptional regulator CysB, thus promoting expression of sulfate acquisition genes [97,107,109]. Expression of sulfate acquisition and reduction genes increases the concentration of sulfur within the cell, completing the loop with high sulfur availability promoting CSC stability and therefore increasing OAS production.

## Development of inhibitors for the OASS isoforms CysK and CysM

### Peptide inhibitors

Salsi et al. (2010) and Spyrakis et al. (2013) have paved the way in CysK inhibitor discovery with the identification of multiple potent peptide inhibitors for several key CysK isoforms [100,110]. Although, more recently, CysK inhibitor studies have focused primarily on chemical inhibitors, these peptide inhibitors stand as key templates for chemical inhibitor designs. The natural inhibition of CysK by CysE has been routinely used for these peptide inhibitor studies as a design platform [6,100,110,111]. The three C-terminal residues of CysE contribute the strongest interactions with CysK, and therefore a minimum three-residue peptide is required for CysK inhibition; both Salsi et al. (2010) and Spyrakis et al. (2013) screened pentapeptides—including an additional two residues to better mimic the full-length CysE C-terminal peptide [100,110,111]. Salsi et al. (2010) utilized the *Hi*CysK crystal structure (1Y7L) in complex with the *Hi*CysE C-terminal peptide [99], replacing this peptide with a panel of 400 pentapeptides into the active site via virtual screening [100]. Spyrakis et al. (2013) followed through an analogous computational pipeline, with the inclusion of the *S. typhimurium* apo CysK (*St*CysK) (1OAS) [93] and *St*CysM (2JC3) [90] crystal structures.

**Figure 6:**
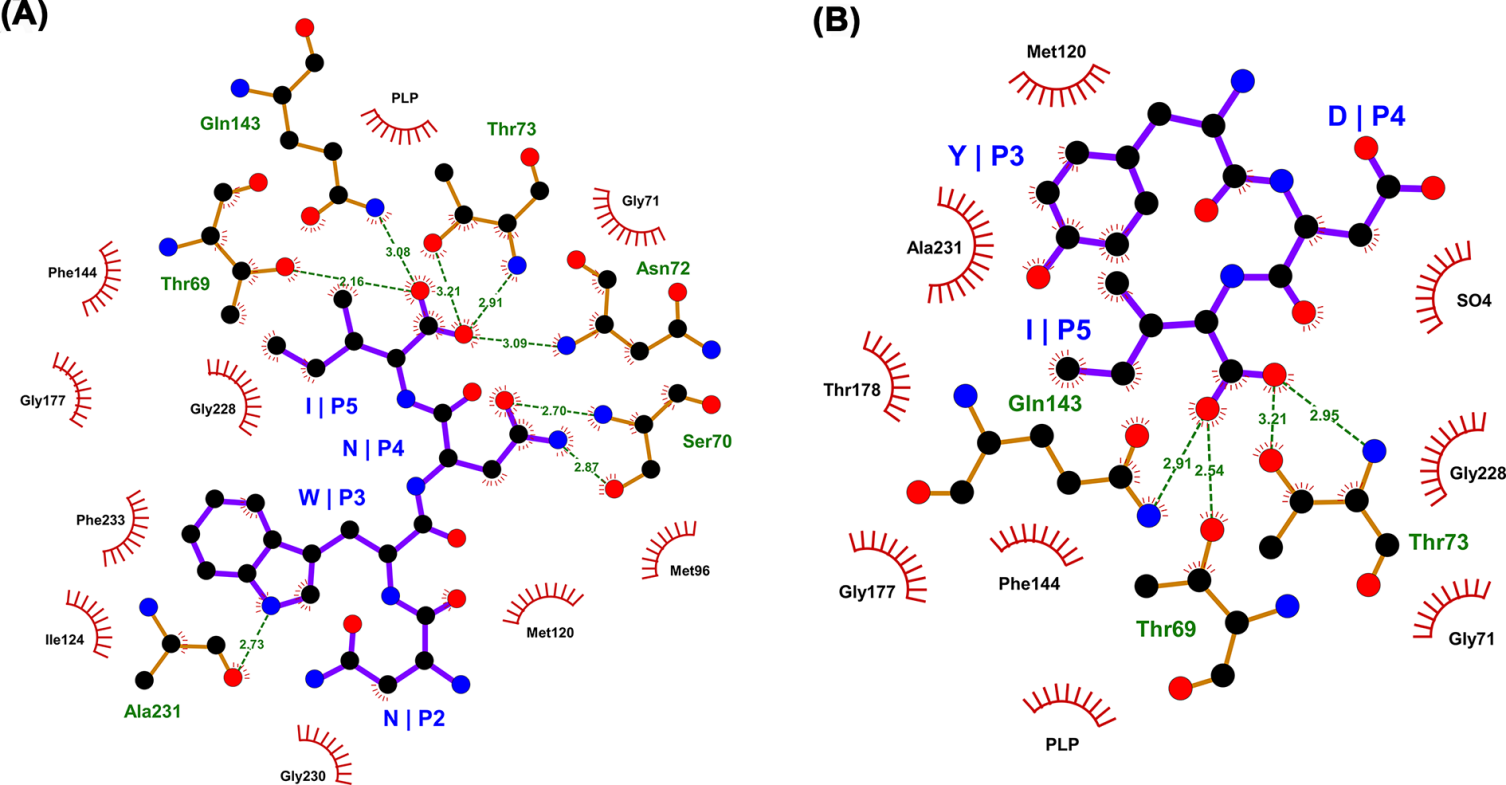
Interaction of MNWNI and MYDI peptides with HiCysK (**A**) LigPlot showing the interactions between the *Hi*CysK residues and the MNWNI pentapeptide (PDB code: 3IQG). (**B**) LigPlot showing the interactions between the *Hi*CysK residues and the MNYDI pentapeptide (PDB code: 3IQH).

Peptide inhibitors for both *S. typhimurium* isoforms and *Hi*CysK were identified, which demonstrated improved potencies *in vitro* compared with their respective native CysE C-terminal pentapeptides (Table 3). The most potent pentapeptides identified for *Hi*CysK were MNWNI and MNYDI, which both exhibited approximately 1.75 times improved affinity for the enzyme in comparison to the equivalent native *Hi*CysE pentapeptide (MNLNI). Interestingly, structural analysis (MNWNI, 3IQG; MNYDI, 3IQH) showed the asparagine in MNWNI at position four to participate in hydrogen-bonding with Ser70; whereas, the aspartate of MNYDI was shown to protrude out toward the protein surface. This protrusion is thought to be a result of the tyrosine at position three participating in an aromatic cluster with Phe144 and Phe233, which allows a sulfate ion to reside within the active site instead, and mimic the interactions of the asparagine in MNWNI (Figure 6) [100].

**Table 3:** List of top characterized CysK peptide inhibitors

Inhibitor	Enzyme	*K*_D_ (µM)^*^	Citation
MNLNI (*Hi*SAT WT)	*Hi*CysK	44.0 ± 3.6	[100]
	*St*CysK	120 ± 12	[110]
	*St*CysM	∼3000^†^	[110]
MNWNI	*Hi*CysK	24.9 ± 3.6	[100]
	*St*CysK	10.4 ± 0.9	[110]
MNYDI	*Hi*CysK	25.8 ± 1.7	[100]
	*St*CysK	0.22 ± 0.04	[110]
YGDGI (*St*SAT WT)	*St*CysK	11.8 ± 0.6	[110]
	*St*CysM	4922 ± 1,030	[110]
YGYDI	*St*CysK	0.42 ± 0.02	[110]
MNDGI	*St*CysK	306 ± 17	[110]
	*St*CysM	1100 ± 100	[110]

* Error reported as standard error.

† No error reported.

Second, and most intriguingly, the top pentapeptide assessed for *St*CysK was MNYDI, which is a pentapeptide based on the *Hi*CysE C-terminus (MNLNI), and not that of the *St*CysE C-terminus (YGDGI) [100]. MNYDI showed approximately 60 times improved affinity compared with YGDGI, and approximately 600 times improved affinity compared with MNLNI. Moreover, the equivalent pentapeptide of MNYDI based on the *St*CysE sequence, YGYDI, still showed reduced potency compared with MNYDI, highlighting that the terminal three residues of the pentapeptides are involved in affinity regulation, whereas the preceding two residues are involved in selectivity [100].

Furthermore, the pentapeptide which demonstrated the greatest potency toward *St*CysM was based on *both* the *St* and *Hi*

CysE C-terminal sequences—MNDGI. This pentapeptide exhibited approximately 4.5 times greater affinity for the enzyme than YGDGI, and around three times greater affinity than MNLNI [100]. This inhibitor is also the most effective against both *S. typhimurium* isoforms, with the difference in *K*_D_ values minimized. Correlation analysis of the *K*_D_ values of *St* isoform binders, demonstrated an inversely proportional relationship, where a more potent inhibitor of *St*CysK was more likely to possess reduced affinity for *St*CysM.

Reasonable affinity correlation was also noted between binders of CysK *H. influenzae* and *S. typhimurium* homologs. Comparison of the active site architecture of these homologs signified only one minor difference—the orientation of the Gln227 side chain. In *Hi*CysK, the R-group protrudes into the active site; whereas, in *St*CysK this group protrudes toward the enzyme surface. Consequently, it is proposed that this localizes the third and fourth residues of the pentapeptides differentially within the enzyme active sites. Nevertheless, this highlights the possibility for synthesizing broad-spectrum CysK and CysM compounds [110].

Altogether, these data demonstrate that effective peptide inhibitors of CysK should ideally possess negatively charged, hydrogen-bond acceptors at position four, and hydrophobic residues at position three. Unfortunately, this trend does not seem to translate to CysM peptide inhibitors, with glycine, a neutral and non-hydrogen bond acceptor at position four, and a negatively-charged, hydrophilic residue at position three.

More recently, Kant et al. (2019) investigated a panel of tetrapeptides for inhibition of the parasite *Leishmania donovani* CysK (*Ld*CysK) [112], with the aim to deconvolute the findings of [113], where *Ld*CysK did not demonstrate a preference for tetrapeptides with either long or small residues. Tetrapeptides were designed to contain all possible amino acid combinations for subsequent docking analysis. Docking analysis revealed EWSI and DWSI as the top two binders, respectively, with EWSI observing greater hydrogen-bonding and hence stabilization capabilities [112]. Therefore, EWSI stands as a starting point for future *Ld*CysK inhibitor designs, alongside the need for *in vitro* characterization. Kant et al. (2019) also followed on to compare the differences between these identified tetrapeptide inhibitors of *Ld*CysK for their affinity for *Mtb*CysK1, given the similarity of these peptides to the native *Mtb*CysE tetrapeptide—DFSI. Interestingly, EWSI demonstrated improved docking into *Mtb*CysK1 compared with both DFSI and EFSI [112]. This highlights EWSI as a valid starting point for future virtual screening of *Mtb*CysK1 chemical inhibitors.

### Chemical inhibitors

Chemical inhibitors stand as superior drug compounds given their improved *in vivo* half-life, bioavailability, and pharmacokinetics compared with peptidic compounds [114,115]. Since the studies by Salsi (2010) was published, many groups have been working on the design, synthesis and characterization of chemical inhibitors for both CysK and CysM. The design work has largely stemmed from structurally mimicking the key chemical groups of the peptide inhibitors. Subsequently, many different chemical inhibitors have been identified, including, cyclopropane carboxylic acids [114,116–119], thiazolidinone and pyrimidinone derivatives [44,120,121], fluoroalanines [122], benzoic acids [7], and pyrazinamines and acetamides [112]. The majority of these compounds were discovered via *in silico* docking experiments using crystal structures of inhibited complexes of the enzymes and subsequent high-throughput virtual screening.

**Figure 7:**
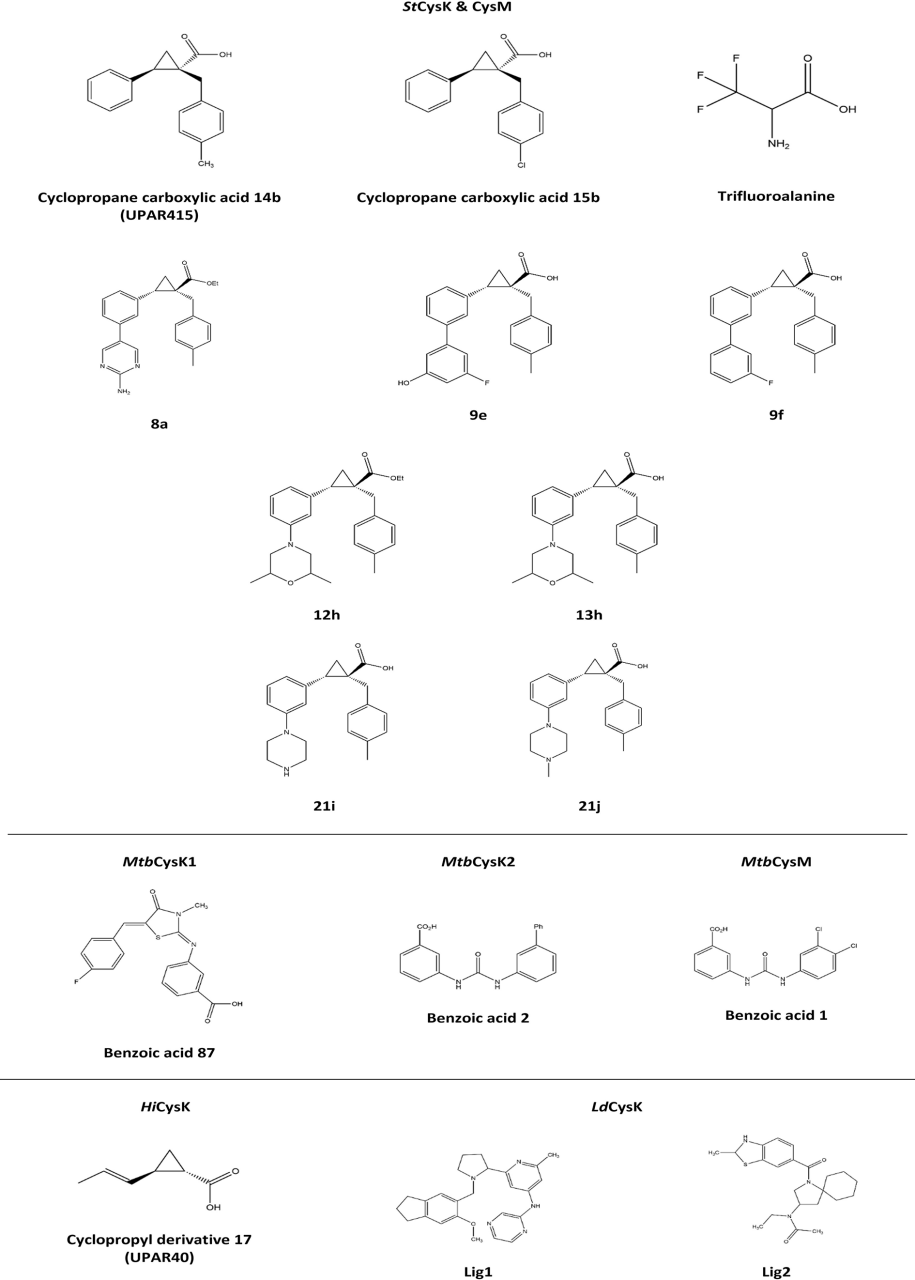
Chemical structures of top OASS chemical inhibitors Figure produced using ChemDraw Prime (RRID:SCR_016768).

To date, Amori et al. (2012) and Pieroni et al. (2016) have discovered the most potent chemical inhibitors of *Hi*CysK - (±)-trans-2-(ethoxycarbonyl) cyclopropanecarboxylic acid (±)-7 (referred to as UPAR40) [114] and trans-2-(prop-1-enyl)-cyclopropanecarboxylic acid (referred to as cyclopropyl derivative 17) [117], respectively. Both compounds contain a carboxylate and a hydrophobic moiety which reflect the key properties of the CysE C-terminal isoleucine, and are incorporated together between a cyclopropane spacer (Figure 7) (cyclopropane spaces are common features in bioactive molecules and also aid in restraining the ligand in its effective conformation for enzymatic interaction) [6,114]. Promisingly, docking and molecular dynamic analyses showed that these inhibitors lock the enzyme in its closed conformation [114,116]. Despite this success, Pieroni et al. (2016) has noted the impracticality of the chemical properties of these compounds for drug-like synthesis [117], and given the inactivity against the CysM isoform, efforts should now be directed toward improving the synthetic feasibility of these compounds, and their activity towards *Hi*CysM, alongside *in vivo* and cytotoxicity assays.

The success of the cyclopropane carboxylic acid compounds for *Hi*CysK, has also been shown for both *S. typhimurium* isoforms. Pieroni et al. (2016) identified (1S,2S)-1-(4-Methylbenzyl)-2-phenylcyclopropanecarboxylic acid 14b and (1S,2S)-1-(4-Chlorobenzyl)-2-phenylcyclopropanecarboxylic acid 15b as the most potent inhibitors against *St*CysM to date (Table 4) These compounds were adapted from cyclopropyl derivative 17 to contain a phenyl group as opposed to a vinyl group (Figure 7), which better embodies the *trans* orientation with the carboxylate displayed by the CysE C-terminal isoleucine, and is more synthetically viable. It was noted that further substitution of cyclopropyl derivative 17 at the α-carbon position for interaction with both *S. typhimurium* isoforms would better occupy a moderately polar area of the active site, and therefore, benzyl substitution at this site with further para substitution resulted in compounds 14b and 15b [117]. Interestingly, the *para* substitution of compounds 14b (4-CH_3_) and 15b (4-Cl) represent both electron withdrawing and donating groups, yet reasonably equivalent potencies are observed (Table 4); however, compound 15b shows reduced selectivity toward either isoform. Promisingly, the IC_50_ values of compound 15b for both enzyme isoforms (Table 4), in the presence of the natural substrates of the enzyme, corroborate with the dissociation constants reported, indicating competitive inhibition.

**Table 4:** Top characterized OASS chemical inhibitors

Inhibitor	Enzyme	IC_50_ (µM)^*^	*K*_D_ (µM)^*^	Citation
Cyclopropyl derivative 7 (UPAR40)	*Hi*CysK	700 ± 53	1.46 ± 0.25	[114]
Cyclopropyl derivative 17		ND	1.45†	[117]
Cyclopropane carboxylic acid 14b (UPAR415)	*St*CysK	ND	0.028 ± 0.005	[117]
	*St*CysM	ND	0.49 ± 0.05	[117]
Cyclopropane carboxylic acid 15b	*St*CysK	0.099 ± 0.004	0.054 ± 0.008	[117]
	*St*CysM	0.50 ± 0.03	0.42 ± 0.06	[117]
8a	*St*CysK	ND	–	[123]
	*St*CysM	ND	–	[123]
9e	*St*CysK	ND	0.035 ± 0.003	[123]
	*St*CysM	ND	0.61 ± 0.08	[123]
9f	*St*CysK	ND	0.051 ± 0.004	[123]
	*St*CysM	ND	1.45 ± 0.31	[123]
12h	*St*CysK	ND	–	[123]
	*St*CysM	ND	–	[123]
13h	*St*CysK	ND	0.066 ± 0.005	[123]
	*St*CysM	ND	3.37 ± 0.72	[123]
21i	*St*CysK	ND	0.45 ± 0.09	[123]
	*St*CysM	ND	83.8 ± 16.1	[123]
21j	*St*CysK	ND	0.25 ± 0.06	[123]
	*St*CysM	ND	23.6 ± 4.5	[123]
Trifluoroalanine	*St*CysK	130 ± 10	ND	[122]
	*St*CysM	1290 ± 230	ND	[122]
Benzoic acid 87	*Mtb*CysK1	0.019 ± 0.0011	ND	[121]
Benzoic acid 1	*Mtb*CysM	ND	0.32 ± 0.01	[7]
Benzoic acid 2	*Mtb*CysK2	ND	22.6 ± 2.4	[7]

ND = not determined.

* Error reported as standard error.

† No error reported.

**Figure 8:**
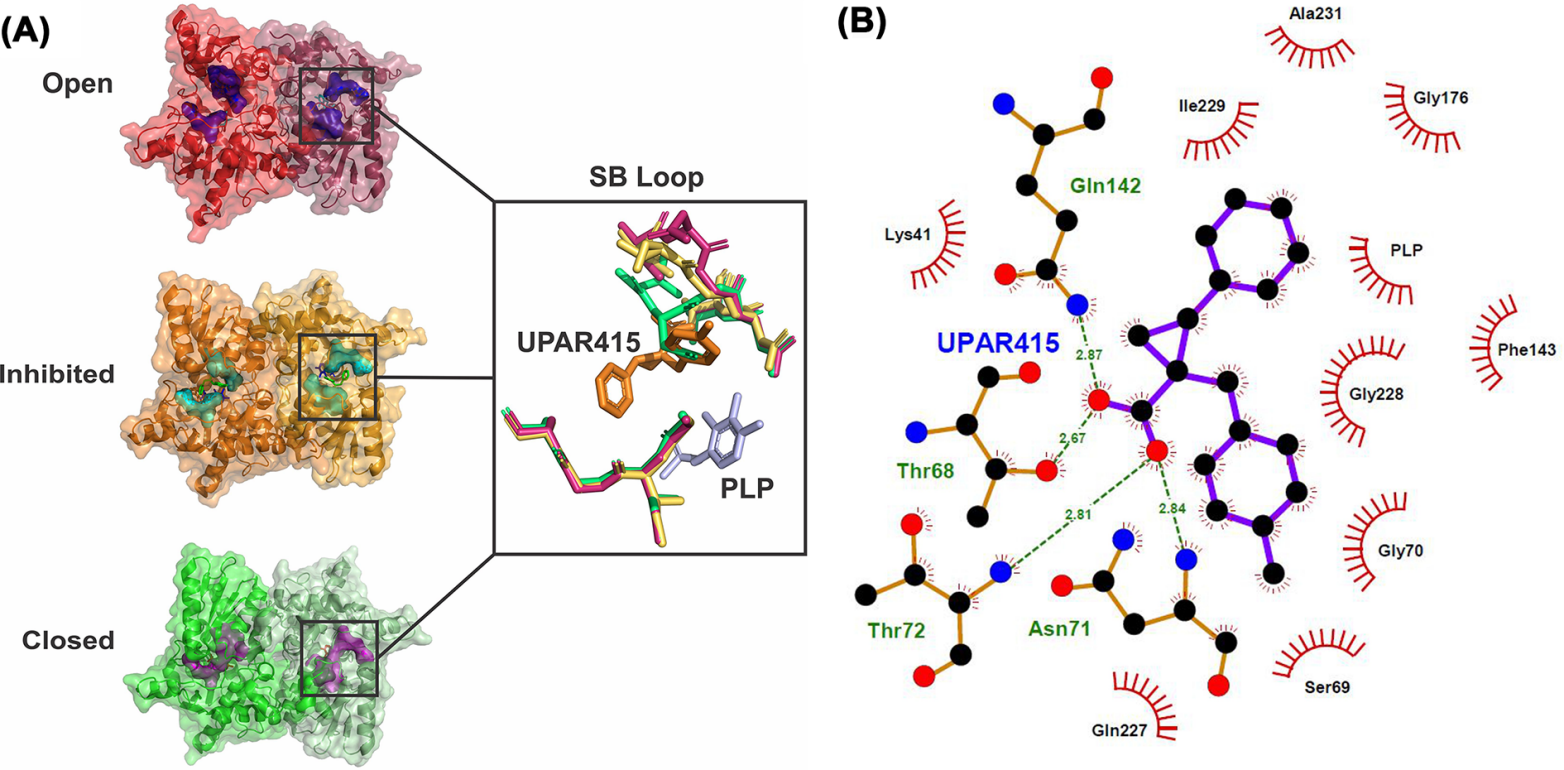
Inhibition of S*t*CysK by UPAR415 (**A**) Structural comparison of the substrate binding loop between the open conformation (1OAS) shown in shades of red, the inhibited conformation (6Z4N) shown in shades of orange, and the closed conformation (1D6S) shown in shades of green. (**B**) LigPlot showing the interactions between the enzyme residues and the UPAR415 molecule. Figure produced using PyMOl and LigPlot.

Recently, Annunziato et al. (2021) utilized compound 14b, here referred to as UPAR415, as an effective adjuvant for the polymyxin antibiotic, colistin [118]. Interestingly, in the presence of low cysteine levels, administration of UPAR415 alone did not exhibit any bactericidal or bacteriostatic effects on multiple bacterial species (Gram-positive and -negative) [118]. In contrast, when UPAR415 was treated under these same conditions in conjunction with colistin, significant deductions were seen in the MIC of colistin compared with when colistin is dosed on its own. Promisingly, the cytotoxicity of UPAR415 was also shown to be insignificant. The crystal structure of *St*CysK in complex with UPAR415 (6Z4N) was also solved by Annunziato et al. (2021). This demonstrated that UPAR415 is a competitive inhibitor of *St*CysK against its first substrate, *O*-acetylserine, and was found localized in proximity to the PLP cofactor (Figure 8). The active site entrance could be seen to be partially blocked by the two aromatic substituents of the cyclopropane-ring, which engaged in hydrophobic interactions with active site residues. The remainder of the UPAR415 molecule was seen to penetrate into the active site. Promisingly, the carboxylate group of UPAR415 was found to localize to the well-studied carboxylic site; engagement with this site has been found to result in a conformational change of the enzyme into its closed active site state through translocation of the substrate-binding loop, which ultimately rotates the N-terminal domain over the active site. Although, UPAR415 positions itself at this carboxylic site, only partial closure of the active site is induced, which can be explained by a steric clash of the substrate-binding loop with the tolyl substituent of UPAR415 (Figure 8) [118].

A medicinal chemistry campaign to synthesize UPAR415 derivatives and the characterization of ligand–target complexes revealed the presence of an accessory sub-pocket that can be filled by substitutions at the 3′ position of UPAR415 [123]. Most compounds synthesized in this study retained good binding *in vitro*. Derivatives with heteroaliphatic or heteroaromatic groups in the 3′ position led improved inhibition against both OASS isoforms compared with those previously described [118]. Compounds substituted with a heteroaromatic group at the 3′ position for example, trans-2-(3′-fluoro-5′-hydroxy-[1,1′-biphenyl]-3-yl)-1-(4-methylbenzyl)cyclopropane carboxylic acid (compound 9e) and trans-2-(3′-fluoro-[1,1′-biphenyl]-3-yl)-1-(4-methylbenzyl)cyclopropane carboxylic acid (compound 9f), that had a phenyl substituted with a fluorine and hydroxy group, or a fluorine respectively demonstrated potent inhibition (nanomolar range) against CysK (Figure 7) [123]. Whereas compounds carrying a 2-aminopyrimidine group at the 3′ position showed good inhibition of both isoforms. Compound 13h (trans-2-(3-(2,6-dimethylmorpholino)phenyl)-1-(4-methylbenzyl)cyclopropanecarboxylic acid) had a dimethyl morpholine at the 3′ position and had the most potent activity for derivatives substituted with a heteroaliphatic group at position 3′ (Figure 7). Compounds with a piperazine ring had good activity against CysK but these derivatives were less effective at inhibiting CysM. The majority of all compounds synthesized demonstrated low toxicity in that they were tolerated by mammalian cells. Compound 13h and its corresponding ester derivative 12 h showed potent inhibition *in vitro* and good toxicity profiles. These lead compounds were tested as colistin adjuvants, showing effective synergy by reducing the MIC of colistin against *E. coli* and *S. typhimurium* even at low concentrations [123]. Importantly for this study, the most promising derivative 12h (trans-ethyl 2-(3-(2,6-dimethylmorpholino)phenyl)-1-(4-methylbenzyl)cyclopropanecarboxylate) was linked to the chemical inhibition of CysK and CysM *in vivo* using target engagement experiments in *S. typhimurium* in the presence of colistin (the compound on its own does not exert any bactericidal effects) [123]. Compound 12h demonstrated a significant improvement in the active concentration at which it can act as a colistin adjuvant inside cells, thereby paving the way as a prodrug to overcome some of the toxicity issues associated with colistin.

Franko et al. (2018) studied fluoroalanine derivatives *in vitro* against both *S. typhimurium* isoforms as irreversible inhibitors, given the ubiquitous use and study of these halogens as irreversible inhibitors of PLP-dependent enzymes [124–132]. Halogenated alanine addition to this group of enzymes is known to generate an unsaturated α-aminoacrylate Schiff's base (or α-aminoacrylate-PLP complex) [127,133]—the subsequent inhibition mechanism is dependent on the specific halogenated alanine and how this reorientates the active site; typically, the catalytic lysine is attacked, and subsequent chemical rearrangement culminates in the disengagement of the halogen ions, allowing for a stable derivative to form, which ultimately inhibits the enzyme [122].

*β*,*β*,*β*-Trifluoroalanine (triF-Ala), which is a known PLP-dependent enzyme suicide inhibitor [125–128,134–136], was the most potent fluoroalanine derivative assessed. This compound had high affinity for the StCysK enzyme active site and slightly lower for that of *St*CysM. The mechanism of inhibition was determined to be irreversible covalent modification of the catalytic amino acids, yet the IC_50_ values of triF-Ala with both enzyme isoforms (Table 4) are too high to be an efficient inhibitor.

Poyraz et al. (2013) identified the most potent inhibitor to date for the *M. tuberculosis* enzyme CysK1 (*Mtb*CysK1), a thiazolidinone derivative - 3-((Z)-((Z)-5-(4-fluorobenzylidene)-3-methyl-4-oxothiazolidin-2-ylidene)amino)benzoic acid [121]. E-pharmacophore sites were identified from the *Mtb*CysK1-DFSI crystal structure (2Q3C) [86]—one aromatic ring, two acceptors, and two negative ionizable moieties. The compound benzoic acid 87 was identified from substitution analyses from the initial *in vitro* hit compound -3-({5-[2-(carboxymethoxy)benzylidene]-3-methyl-4-oxo-1,3-thiazolidin-2-ylidene}amino)benzoic acid 2, where compound 87 represents a C4 fluoro substitution, and showed an approximately five fold greater inhibition effect compared to benzoic acid 2.

The crystal structure of derivative 2 bound to *Mtb*CysK1 (3ZEI) showed the thiazolidinone moiety to not protrude greatly out of the active site as is seen with the native peptide [121]. The thiazolidine core mimics the phenyl group of the DFSI peptide and contributes to interactions with the enzyme hydrophobic cleft. Although similarly, the carboxylic moiety of the compound benzoic acid 2 (associated with the benzoic acid group) was found docked entirely within the active site with Ser72 interactions and potential for hydrogen-bonding with Thr71 and Gln144, as has previously been shown with the C-terminal isoleucines of peptide inhibitors [100,110]. In contrast with previous inhibitor observations, the 2-carboxymethoxy moiety does not participate in any solvent hydrogen-bonding despite protruding out toward the protein surface, which may indicate a key feature in developing future potent inhibitors [6,121]. Nevertheless, this structural analysis revealed an enzyme pocket next to the para-position of the benzylidene ring, from which compound 87 verifies [121].

Brunner et al. (2016) identified the most potent inhibitor to date for *Mtb*CysM - 3-(3-(3,4-dichlorophenyl)ureido) benzoic acid 1, as well as that for *Mtb*CysK2 - 3-(3-([1,1-Biphenyl]-3-yl)ureido)benzoic acid 2 [7]. These hit compounds were identified by *in vitro* screening of approximately 17,000 small molecules, followed by structural analysis. The crystal structure of the compound benzoic acid 1 bound to *Mtb*CysM (5I7A) did not demonstrate large differences compared with the apo structure—the compound was shown to be bound within the open state of the active site (parallel to PLP pyrimidine ring plane), where stacking interactions were seen between the PLP pyrimidine ring and the urea moiety [7]. The meta carboxylate group of the compound was found associated within the active site, similar to the carboxylate moiety of the α-aminoacrylate intermediate of *Mtb*CysK1 [86]; whereas the 1,2-dichlorobenzene group interacts within the hydrophobic cleft of the enzyme. The core urea group was found to interact with the Asn221 side chain via its carbonyl moiety, interactions with the carboxyl of Ala323 found within the active site occur through an amide group, with the other amide group forming a hydrogen-bond with a water molecule [7]. Promisingly, in a nutrient starvation model (simulating dormancy, when CysM is primarily expressed), compound 1 demonstrated higher potency compared with current clinically approved first-line tuberculosis antibiotics, with insignificant cytotoxic effects on several mammalian cell lines [7].

Kant et al. (2019) identified two hit molecules for *Ld*CysK - N-(2-{1-[(6-methoxy-2,3-dihydro-1H-inden-5-yl)methyl]pyrrolidin-2-yl}-6-methylpyridin-4-yl)pyrazin-2-amine (Lig1) and N-ethyl-N-{1-[(2-methyl-2,3-dihydro-1,3-benzothiazol-6-yl)carbonyl]-1-azaspiro[4.5]decan-3-yl}acetamide (Lig2) via *in silico* screening of tetrapeptides with shape similarity to EWSI and DWSI [112]. These ligands were docked into *Ld*CysK and Lig2 demonstrated improved interaction energy and capacity for hydrogen-bonding; this ligand was that which had greater conformational similarity to EWSI compared to DWSI. This potency of Lig2 was reinforced through molecular dynamics and binding energy analysis compared to Lig1 in complex with the enzyme, in terms of stability and compactness. In addition, both docking and molecular dynamic analyses demonstrated hydrogen-bonding interactions between Lig2 and residues, Ser79 and Gln152 [112]. Characterization of these inhibitors both *in vitro* and *in vivo* remains to be investigated.

It is worth noting that despite the significant advances that have been made in the chemical inhibitor space for CysK and CysM enzymes, the potencies of these inhibitors remain around 100-fold less effective than the complete enzymatic inhibition of CysK by CysE (Table 2).

## Conclusion

There have been many campaigns aimed at discovering potent and selective inhibitors for the cysteine synthesis enzymes CysE and CysK. Yet despite excellent inhibition of activity seen for certain compounds many failed to inhibit bacterial growth, presumably due to the lack of permeability of the compounds across the bacterial wall. The best inhibitor for CysE that was bactericidal and had an IC_50_ of 48 µM, although this is much greater than the natural inhibitor cysteine, with an IC_50_ between 1 and 10 µM. Recently inhibitors of CysK were identified that demonstrate potent inhibition (nanomolar binding) and an adjuvant effect when used in combination with the antibiotic colistin. Based on this it is worth testing other promising inhibitors identified with known antibiotics to see if they have an adjuvant effect. Given that cysteine biosynthesis is often dispensable under nutrient rich conditions but becomes more essential during infection and persistence it would also be worth testing if the identified compounds reduces infection and/or enhances clearance by the host immune system. As shown here there is increasing evidence for bacterial *de novo* cysteine biosynthesis as a promising drug target for either new antimicrobials or antibiotic adjuvants. Further validation of this pathway and further exploration of new and existing inhibitor compounds is vital to develop potent and selective inhibitors to overcome antimicrobial resistance in a range of Gram-positive and Gram-negative human pathogens.

## Abbreviations

acetyl-CoA: acetyl coenzyme A
AMR: antimicrobial resistance
CysE: serine acetyltransferase
MDR: multidrug resistant
MIC: minimum inhibitory concentration
OAS: *O*-acetylserine
OASS: *O*-acetylserine sulfhydrylase
